# Independent Tumor Origin in Two Cases of Synchronous Bilateral Clear Cell Renal Cell Carcinoma

**DOI:** 10.1038/srep29267

**Published:** 2016-07-07

**Authors:** Zhengguo Ji, Jialu Zhao, Tian Zhao, Yuying Han, Yujun Zhang, Haihong Ye

**Affiliations:** 1 Department of Medical Genetics and Developmental Biology, School of Basic Medical Sciences, Capital medical University, Beijing, China; 2 Department of Urology, Beijing Friendship Hospital, Capital medical University, Beijing, China; 3 Beijing Institute for Brain Disorders, Center of Schizophrenia, Capital Medical University, Beijing 100069, China; 4 Institute of Chinese Materia Medica, Chinese Academy of Chinese Medical Science, Beijing 100700, China

## Abstract

Bilateral renal cell carcinomas (RCCs) pose a challenge for clinical treatment and management. Most bilateral RCCs are sporadic, and do not show a hereditary pattern indicative of VHL syndrome or other inherited cancers. The origin and evolution of these sporadic bilateral RCCs remains elusive. We obtained normal and tumor samples from two male patients suffering from early stage synchronous bilateral clear cell RCC (ccRCC), and analyzed genomic DNA using whole exome sequencing and bisulfite pyrosequencing. We detected distinct 3p loss of heterozygosity (LOH) in both tumors in each patient. Two tumors within the same patient harbored distinct driver mutations and different CpG hypermethylation sites in the *VHL* promoter. Moreover, tumors exhibit independent evolutionary trajectories. Therefore, distinct 3p LOH, combined with contingent driver gene mutations and independent *VHL* hypermethylation, led to independent tumor origin and parallel evolution of bilateral ccRCC in these two patients. Our results indicate that tumors in these two cases were not due to common germline oncogenic mutations. They were results of multiple *de novo* mutations in each kidney, rather than primary ccRCC with contralateral renal metastasis. Therefore, histopathologic and genetic profiling from single tumor specimen may underestimate the mutational burden and somatic heterogeneity of bilateral ccRCCs.

Renal cell carcinoma (RCC) accounts for 2–3% of all adult malignancies^1^,^2^. RCC can be histologically classified into a few subtypes, and clear cell renal cell carcinoma (ccRCC) constitutes 70–80% of all RCCs^1^. In most cases RCCs occur in one kidney (solitary RCC), and migrate to other organs at advanced stages. However, in a small set of patients (0.7–4.7%), RCCs occur in both kidneys (bilateral RCC)^3^,^4^. Synchronous bilateral RCC (diagnosed concomitantly or within 6 months of the first primary tumor) was reported to occur in 0.3–2.6% RCC patients^3^,^5^. Metachronous bilateral RCCs (diagnosed at least 6 months after the first primary tumor) developed in about 0.4% RCC patients^3^,^6^. Similar prevalence was also observed in a Chinese population^7^. Most bilateral RCCs are sporadic, and do not show a hereditary pattern indicative of Von Hippel-Lindau (VHL) syndrome, hereditary papillary renal carcinoma, or other inherited cancer^3^. The origin and evolution of these sporadic bilateral RCCs still remain elusive. Because of the vital function of kidneys, partial nephrectomy is preferred for these patients, as radical nephrectomy of both kidneys would result in life-long hemodialysis^1^,^8^. In this circumstance, reducing recurrence rate and potential invasiveness poses a challenge. Therefore, understanding tumorigenesis of bilateral RCC is much relevant to clinical treatment and management of this cancer.

In recent years, studies using integrated and comprehensive genomic profiling of ccRCCs have identified critical genetic and epigenetic events involved in this cancer. The majority of known events are frequently subclonal, while *VHL* mutation/hypermethylation and Chromosome 3p loss of heterozygosity (3p LOH) are the only consistently shared events in most ccRCCs^9^. Therefore, 3p LOH and *VHL* mutation/hypermethylation are considered two predominant founder drivers for sporadic ccRCC^10^,^11^,^12^,^13^,^14^,^15^,^16^,^17^,^18^. *VHL* gene is located on the short arm of chromosome 3. 3p LOH and silencing of the remaining *VHL* allele, either by somatic mutation or promoter hypermethylation, lead to complete inactivation of *VHL*. Besides *VHL*, three more ccRCC driver genes, *PBRM1*, *BAP1* and *SETD2*, are also located between 3p25 and 3p21, the common site of 3p LOH in ccRCCs^12^,^14^.

Multiple renal cysts and carcinomas in both kidneys are common in patients with VHL syndrome. VHL syndrome is found in 4.3% of bilateral RCCs^19^. The hereditary VHL syndrome is attributed to a germline mutation of one allele of the *VHL* gene, followed by loss of the remaining wild-type allele sometime during the carriers’ life^20^. However, the majority of bilateral RCC patients are sporadic. It remains to be elucidated whether a common germline mutation leads to the development of sporadic bilateral RCCs, or two tumors are completely independent. The aim of this study was to investigate the origin and evolution of synchronous bilateral ccRCC through whole exome sequencing (WES) and bisulfite pyrosequencing of samples from two such cases.

## Results

### Whole exome sequencing (WES)

**Figure 1:**
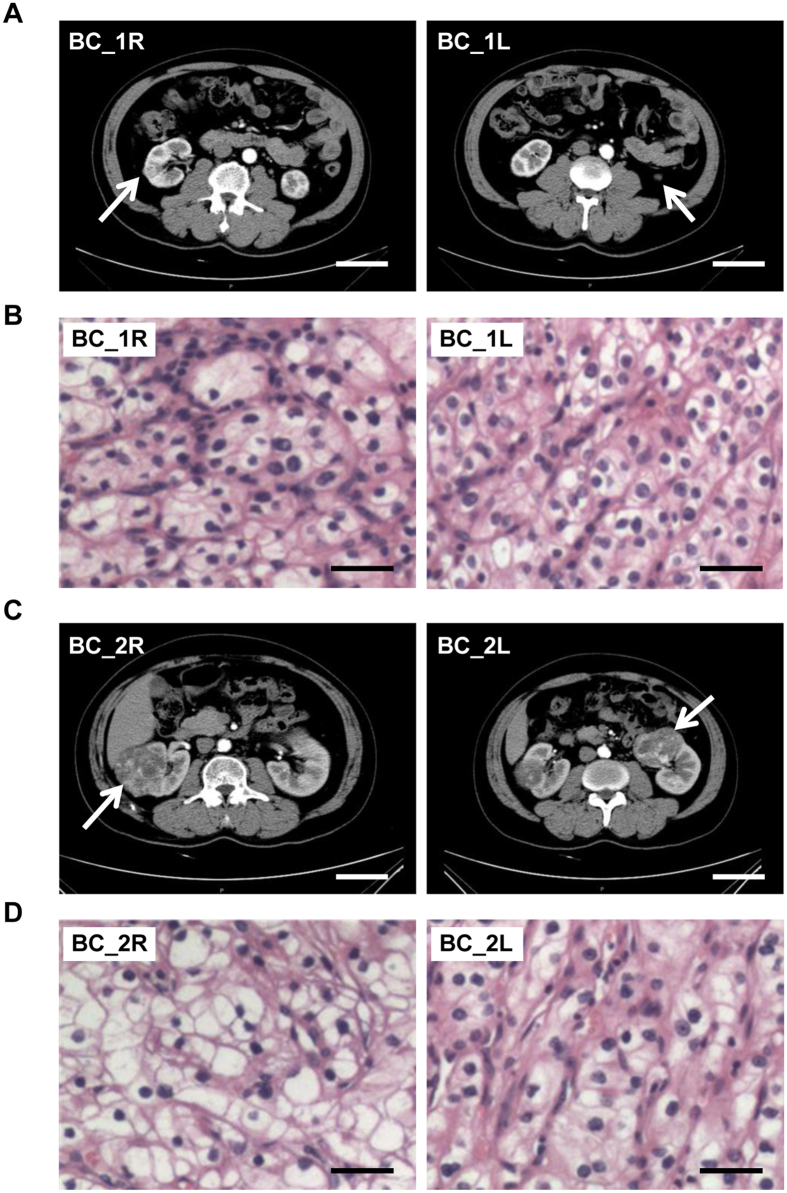
Imageology and histopathology information of the two synchronous bilateral ccRCC patients. (**A**) CT scan shows the tumor size and perinephric circumstance of two kidneys in case BC_1. (**B**) Hematoxylin and eosin (HE) staining of bilateral ccRCC in case BC_1. (**C**) CT scan images of bilateral ccRCC in case BC_2. (**D**) HE staining of bilateral ccRCC in case BC_2. Arrow, tumors. Scale bars in CT scan, 5 cm; scale bars in HE staining, 10 μm.

Two male patients (BC_1 and BC_2) suffering from early stage synchronous bilateral ccRCC were involved in this study (Supplementary Table 1 and Fig. 1). Both tumors from patient BC_1 were classified as stage I (pT1aN0M0) according to the TNM classification of renal cell carcinoma, while tumors in patient BC_2 were slightly more advanced (pT1bN0M0) (Supplementary Table 1)^21^,^22^.

For genetic analysis, one region with homogenous macroscopic appearance from each tumor was chosen for WES. We also harvested peritumoral tissues (normal kidney tissue) for WES, and used it as reference to separate somatic mutations from germline variants in tumors. From patient BC_1, tumor samples BC_1R and BC_1L were taken from right and left kidney, respectively, whereas BC_1P was taken from normal peritumoral tissue in the right kidney. Similar nomenclature was applied to patient BC_2.

WES was performed to an average depth of 50× or greater in all of these samples. Over 80% of targeted bases were covered by at least 10 reads. A total of 413 exome variations consisted of 360 single nucleotide variants (SNVs) and 53 insertion/deletions (indels) were detected in patient BC_1. There were 401 exome variations consisted of 352 SNVs and 49 indels in patient BC_2 (Supplementary Table 2). To validate the WES data, PCR and Sanger sequencing were performed for 11 variant alleles (including SNVs and indels) detected in driver genes or significantly mutated genes in ccRCC, each in all three samples in the corresponding patient. One mutation in *NAV3* in sample BC_2L was not validated, and the estimated validation rate is 97% (Supplementary Table 3).

### Distinct 3p LOH events in tumors from both bilateral ccRCC patients

**Figure 2:**
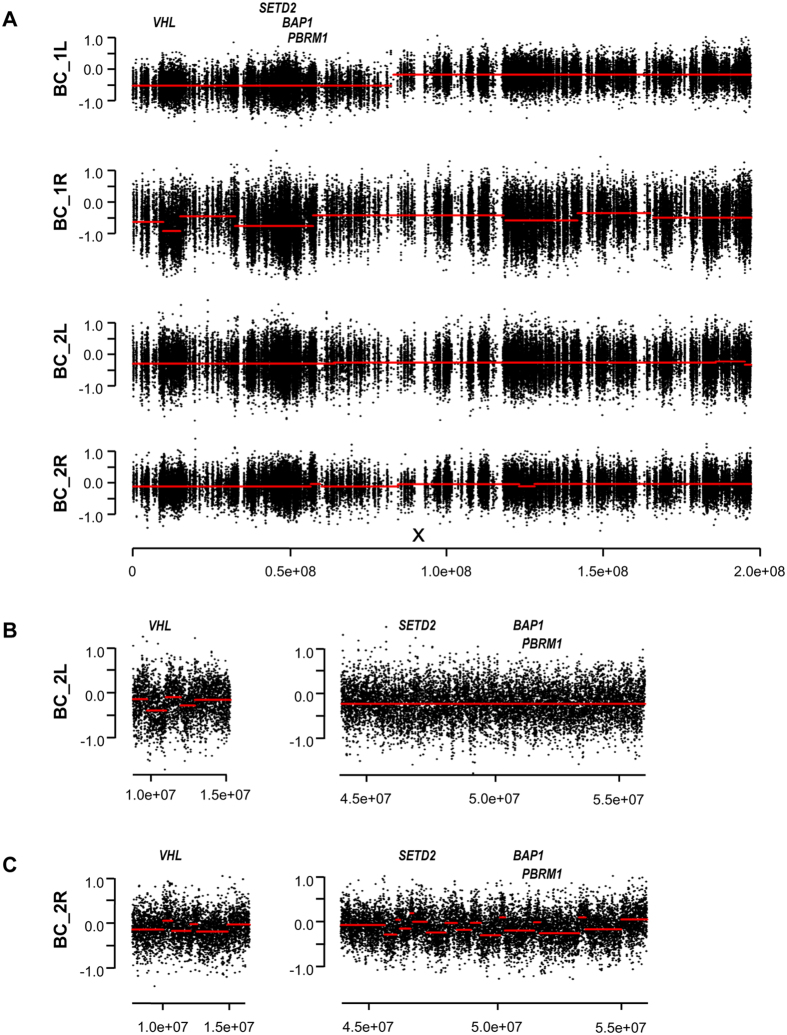
Somatic copy number analysis of chromosome 3 in four tumors. (**A**) Copy number profiles of chromosome 3 of four tumors. (**B**,**C**) Focal copy number profiles covering ccRCC driver genes on chromosome 3p. Y-axis is log_2_R, and the X-axis represents position along chromosome 3 in hg19. X indicates the approximate position of the centromere. Redlines, the average Log_2_R of variant alleles calculated using a circular binary segment (CBS) algorism, are indicative of the copy numbers of the corresponding chromosomal segments.

As 3p LOH is one of the founder events in the origin of ccRCC, we sought to identify possible LOH events in critical regions by analyzing somatic copy number aberration (SCNA) profiles reconstructed from the WES data. Indeed, both tumors from patient BC_1 displayed 3p LOH, although the profiles are different. For the left tumor (BC_1L), there was a single breakpoint mapped to 3p12.2 (close to pseudogene *CYP51A1P1*) (Fig. 2A). For tumor BC_1R, two segments of SCNAs were present in the chromosome 3p. One segment covered *VHL* (3p25.3-3p25.1, from *TTLL3* to *CAPN7*), while the other spanned ccRCC driver genes *SETD2*, *PBRM1* and *BAP1* (3p22.3-3p14.3, from *CNOT10* to *DENND6a*) (Fig. 2A). For patient BC_2, no large scale SCNA segments were observed in chromosome 3; however, many focal events were detected instead (Fig. 2). Moreover, these two tumors exhibited distinct focal SCNA profiles. In tumor BC_2R, focal SCNAs were present in regions covering *VHL*, *SEDT2*, *PBRM1* and *BAP1*, while focal SCNAs were only detected in the region covering *VHL* in the left tumor (BC_2L) (Fig. 2B,C).

Besides chromosome 3, SCNAs were also detected in other chromosomes in BC_1. There were large scale SCNA segments in chromosome 16, 20 and 22 in tumor BC_1R, and loss of one copy of whole chromosome 6 and 14 in tumor BC_1L (Supplementary Figure 1). No large scale SCNA segments were observed in tumors from patient BC_2 (Supplementary Figure 2).

### Distinct *VHL* mutations and hypermethylation sites in its promoter region

**Figure 3:**
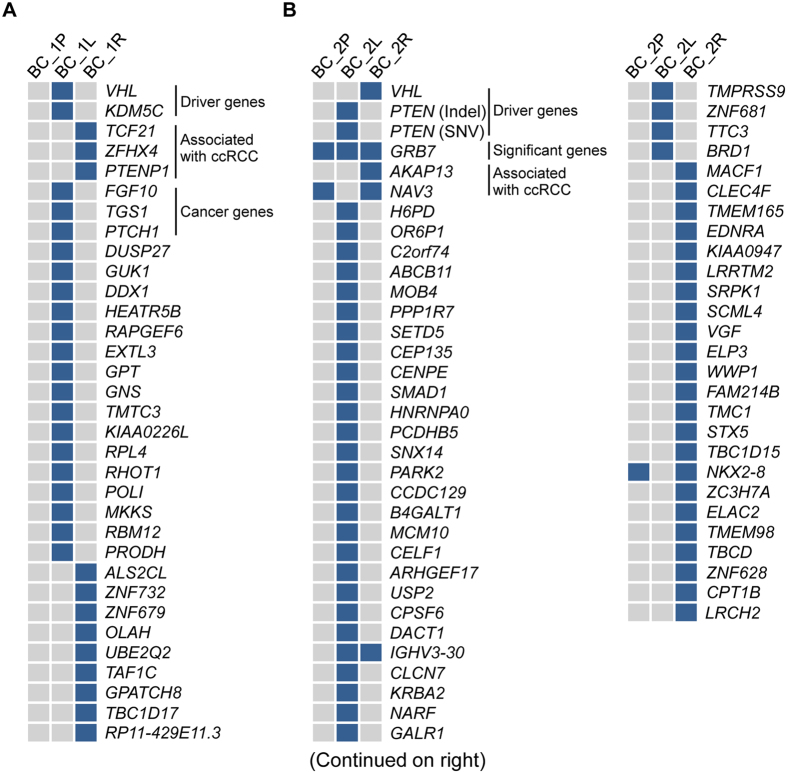
Non-synonymous and splicing site somatic mutations in four tumors. (**A**) Mutations in case BC_1. (**B**) Mutations in case BC_2. Blue indicates the presence of mutations.

**Figure 4:**
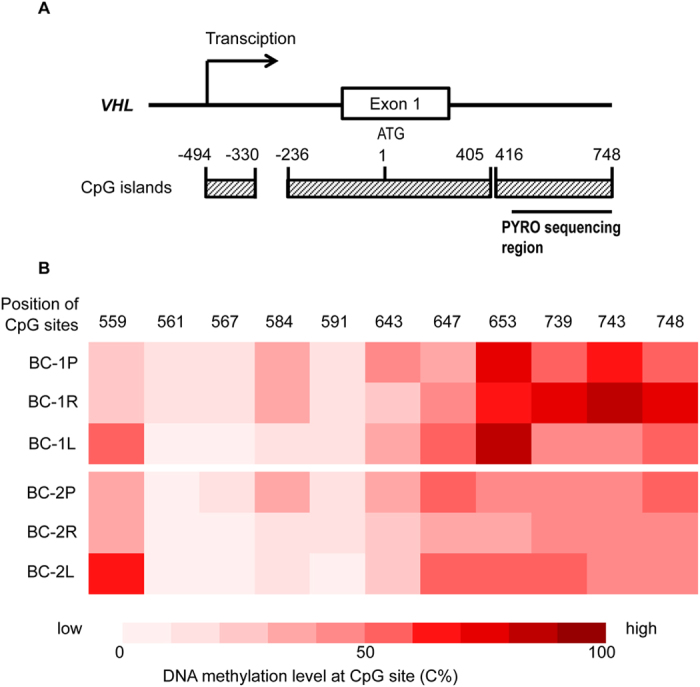
DNA methylation level of CpG sites in the *VHL* promoter region. (**A**) Diagram of the CpG islands in the *VHL* promoter region. The first A of the translation starting codon in exon 1 was designated as Position 1. (**B**) DNA methylation level of 11 CpG sites presented as C% in Pyrosequencing data.

We next examined *VHL* gene for possible mutations from our WES data. For both patients, no non-synonymous mutations were detected in *VHL* in the normal peritumoral tissues, which were confirmed via Sanger sequencing of all 3 exons and splice sites of this gene. This result indicates that unlike VHL syndrome, germline mutation in *VHL* gene was not the cause of bilateral ccRCCs in these two patients. Interestingly, a deleterious frameshift deletion in *VHL* was present in tumor BC_1L, but no *VHL* mutation was detected in tumor BC_1R or in the normal peritumoral tissue (BC_1P) (Fig. 3 and Supplementary Tables 4 and 5). The promoter region of *VHL* gene in both tumors was hypermethylated; however, the CpG sites with high methylation level were different between two tumors (Fig. 4). In patient BC_2, a deleterious frameshift deletion in *VHL* was present in tumor BC_2R, while CpG sites with high methylation level were detected in the *VHL* promoter in tumor BC_2L (Figs 3 and 4). All *VHL* mutations were confirmed via Sanger sequencing (Supplementary Table 3). Therefore, tumors in these two patients with synchronous bilateral ccRCCs did not share common *VHL* mutations.

Interestingly, in two patients with metachronous bilateral ccRCCs (patients BC_3 and 4), we also observed distinct *VHL* mutations from Sanger sequencing results (Supplementary Tables 1 and 6). Similar to the two synchronous bilateral ccRCC cases, no germline *VHL* mutation was observed. In patient BC_3, an SNV with a deleterious effect on protein function (PROVEAN score = −4.53) in exon 2 was detected in 4 different regions in the right tumor, while a frameshift deletion in exon 3 was detected in the left tumor. In patient BC_4, a frameshift insertion in exon 1 was present in the left tumor, while no *VHL* mutation was detected in two different regions of the right tumor examined (Supplementary Table 6).

### Distinct non-synonymous somatic mutations and evolutionary trajectories

We next analyzed other non-synonymous and splicing site somatic mutations in tumors delineated from our WES data (Supplementary Table 4). Genes were assigned into five categories according to published criteria: 1, driver genes of ccRCC; 2, significantly mutated genes in ccRCC; 3, genes with mutations associated with ccRCC; 4, other cancer genes; 5, genes of unknown significance to cancers^12^,^13^,^14^,^23^. Two tumors from patient BC_1 harbored different mutations in genes from all five categories (Fig. 3). No deleterious mutations in driver genes except *VHL* were present in tumors of BC_1. The SNV present in the driver gene *KDM5c* in BC_1L only has a neutral effect on its protein function ((PROVEAN score = −1.77, Supplementary Table 5). Therefore, 3p LOH and frameshift deletion/hypermethylation in *VHL* led to tumorigenesis of tumor BC_1L, while LOH at 3p segment covering *VHL* and *VHL* promoter hypermethylation were two founder drivers for tumor BC_1R.

In patient BC_2, tumor BC_2L harbored a deleterious frameshift deletion in *PTEN*, another driver gene of ccRCC. Interestingly, a non-synonymous SNV was present in *GRB7*, a significantly mutated gene in ccRCC, in both tumors and in the peritumoral tissues (Fig. 3). However, the predicted neutral effect of this SNV (PROVEAN score = −2.78) suggests that it is unlikely to have an effect on its protein function, ruling out its possible role as early founder mutations for bilateral ccRCCs in this patient (Supplementary Table 5). Together, these data suggest that tumors in patient BC_2 did not share common founder driver mutations.

Non-synonymous and splicing site mutations in genes of unknown significance to cancer also differed between two tumors in patient BC_1 (25 genes in total, Fig. 3A). In patient BC_2, only one out of 54 mutations in genes from this category was shared between two tumors (Fig. 3B). This gene, *IGHV3–30*, encodes the immunoglobulin heavy chain variable region and has no known association with tumorigenesis of ccRCC and its related pathways.

**Figure 5:**
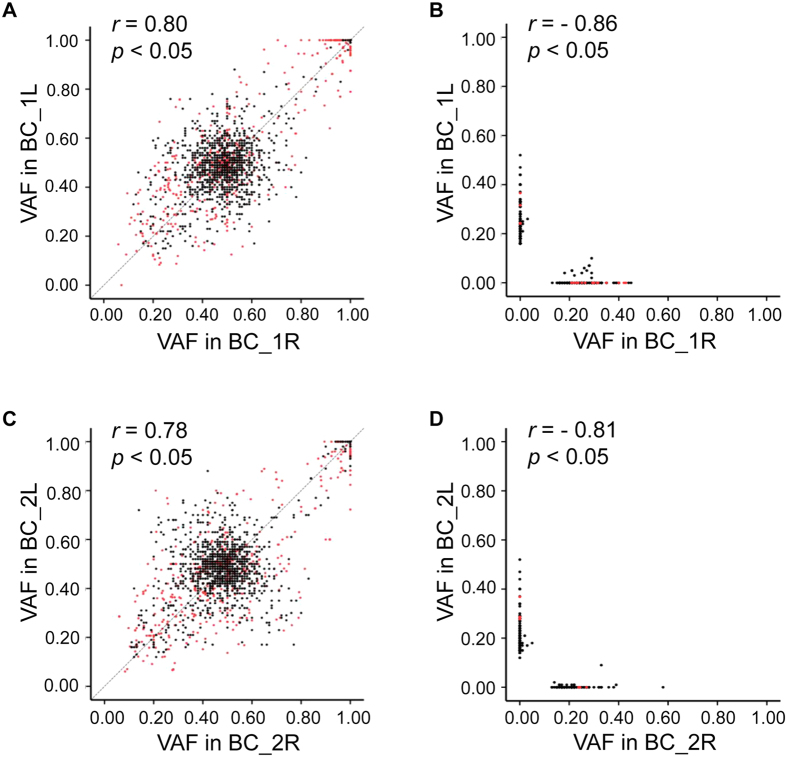
Variant allele frequency analysis of all mutations in four tumors. (**A**,**B**) Mutations in case BC_1. (**C**,**D**) Mutations in case BC_2. (**A**,**C**) Germline variants. (**B**,**D**) tumor-specific mutations. Each dot represents a variant allele detected in WES. The value of each dot is calculated as variant allele depth/total depth. Black dots represent SNVs and red dots represent indels. *r*, Pearson correlation coefficient; *p* < 0.05, statistically significant.

The number of non-synonymous and splicing site somatic mutations in these two patients is small compared to previous reports with larger cohorts and more advanced ccRCCs, presumably due to an early tumor stage. To further compare the evolution of two tumors in the same patients, we analyzed variant allele frequency (VAF) of all variants (excluding known high frequency SNVs and indels in public databases) delineated from the WES data. Changes in allele frequencies happen during cancer evolution due to stochastic processes^13^,^24^. Therefore VAF, calculated by counting the number of Illumina sequencing reads for each variant allele, can be used as an indicator of cancer progression and heterogeneity. For variant alleles shared between tumors and the normal tissue in patient BC_1, VAFs in two tumors are highly correlated (r = 0.80), suggesting their common origins as germline variants (Fig. 5A). However, for tumor-specific variant alleles (not present in the normal tissue), most were detected in only one tumor (237 out of 253 variant alleles have a VAF > 0.1 in one tumor but VAF = 0 in the other). Overall, tumor-specific VAFs are negatively correlated (r = −0.86) (Fig. 5B). Similar pattern of VAFs was also observed in patient BC_2, where 152 out of 169 tumor-specific variants were exclusively observed in one tumor only (r = −0.81) (Fig. 5C,D). Together, these results indicate that two tumors in bilateral ccRCCs have distinct evolutionary trajectories.

### Convergence on the cancer-related pathways

**Figure 6:**
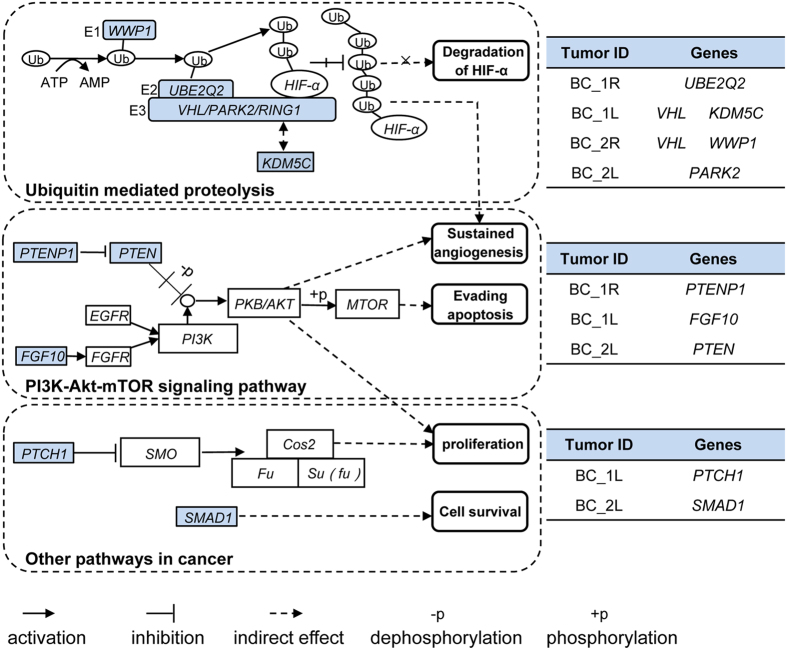
Convergence of non-synonymous and splicing site somatic mutations on ubiquitin-mediated proteolysis pathway, PI3K-Akt-mTOR signaling pathway and other pathways in cancer. Genes with mutations detected in tumor samples are colored blue.

Despite genetic divergence between two tumors in both bilateral ccRCC patients, non-synonymous and splicing site mutations in all four tumors displayed functional convergence on cancer-related pathways, including the ubiquitin-mediated proteolysis pathway, PI3K-Akt-mTOR signaling pathway, and other caner related pathways (Fig. 6 and Supplementary Table 7). For example, tumor BC_1L and tumor BC_2R harbored frameshift deletions in *VHL* whose protein product possesses E3 ubiquitin ligase activity^25^, while protein product of *KDM5C* (mutated in tumor BC_1L) interacts with RING1, an E3 ligase in the ubiquitin pathway. Tumor BC_2R harbored a mutation in *WWP1* that encodes an E1 ligase. Interestingly, although no *VHL* mutation was detected in tumor BC_1R and BC_2L, mutations in *UBE2Q2* and *PARK2*, two genes involved in ubiquitin-mediated proteolysis, were present in these two tumors, respectively.

PI3K-AKT-mTOR signaling pathway is another important pathway involved in ccRCC. Tumor BC_2L contained a frameshift mutation in *PTEN* which encodes a phosphatase that dephosphorylates and inactivates Akt. Tumor BC_1R harbored a mutation in *PTENP1*, a gene located within *PTEN* that encodes a non-coding RNA negatively regulating *PTEN* expression^26^,^27^. A mutation in *FGF10* was detected in tumor BC_1L. *FGF10* is able to activate PI3K-Akt signaling via FGFR. No mutation in genes involved in this pathway was detected in tumor BC_2R.

Besides ubiquitin-mediated proteolysis pathway and PI3K-AKT-mTOR signaling pathway, other genes involved in cancer pathways were also detected. For example, mutation in *PTCH1*, a gene regulating cell proliferation, was present in tumor BC_1L. Tumor BC_2L harbored a mutation in *SMAD1*, a gene regulating cell survival. Despite profound genetic divergence between tumors in bilateral ccRCCs, pathway convergent occurs in cancer progression, indicating that evolutionary selection and adaptation control cancer progression in the presence of a high degree of mutational diversity.

## Discussion

Here we report WES analysis and bisulfite pyrosequencing of two sporadic cases of early stage, synchronous bilateral ccRCCs in the absence of VHL syndrome. For both cases two tumors within the same patient contained distinct driver mutations. Mutations in crucial genes, including *VHL* and *PTEN*, were present in only one tumor in each patient. Different CpG sites in the *VHL* promoter region were hypermethylated in tumor samples from the same patients, indicating independent *VHL* promoter hypermethylation during tumorigenesis. Although 3p LOH happened in both tumors in the bilateral ccRCC patients, they were *de novo*, independent genetic lesions. Taken together, our results suggest that there were no common founder events for the origin of these synchronous bilateral ccRCCs. Occurrence of bilateral ccRCCs is not due to germline mutations or *de novo* somatic mutations early in embryonic development.

Intratumor heterogeneity (ITH) is commonly observed in solitary ccRCCs, especially at more advanced stages. Different regions in solitary ccRCCs with ITH share common founder driver events, 3p LOH and *VHL* mutation/hypermethylation. They harbor different mutations in other genes, including driver gene *SETD2*, *PBRM1* and *BAP1*^13^,^28^,^29^,^30^. The genetic differences between tumors in our cases of synchronous bilateral ccRCCs, however, are not completely due to ITH. In these two cases, two tumors within the same patient did not harber the same founder driver mutations, 3p LOH and *VHL* mutation/hypermethylation, indicating their independent tumor origin. Therefore, it is unlikely that they were primary ccRCC with contralateral renal metastasis. Interestingly, in the metachronous bilateral ccRCC patient BC_3, four regions in the right tumor harbored the same *VHL* mutation, while a different *VHL* mutation was present in the left tumor, suggesting that different regions in the right tumor had a common origin and the left tumor was independent. Whether differences in other gene variants are due to ITH or parallel tumor evolution cannot be clearly delineated from our present WES data; however, they are more likely to be the results of independent tumor evolution considering the independent origin of tumors in these two cases.

Genetic analysis of this rare form of cancer offers a unique opportunity to study tumor evolution. Our results suggest that sporadic synchronous bilateral ccRCC may well be a result of multiple *de novo* mutations in each kidney, rather than common germline oncogenic mutations as in the commonly known bilateral retinoblastoma and VHL syndrome. Distinct somatic mutations converging on a few common important signaling pathways lead to occurrence of RCCs with identical histopathological characteristics. The same organ (kidney) sets histological constraints within a restricted range during origin and evolution of bilateral RCCs. In-depths analysis on more such patients is necessary to confirm our findings. Moreover, it would be interesting to examine cancers in other bilateral organs, including breasts and lungs, to confirm the existence of independent tumorigenesis within one patient.

Our finding that sporadic synchronous bilateral ccRCCs may have independent tumor origin and evolution will have a few important clinical implications for precicsion medicine. These observations also indicate the need for optimizing tumor sampling when evaluating the genomic landscape of ccRCC^9^. First, histopathologic and genetic profiling from single tumor specimens may underestimate the mutational burden and somatic heterogeneity of bilateral cancers. Biopsy of tumors from both sides is necessary, not only for the identification of specific biomarkers, but also for genetic analysis to facilitate clinical decision making in subsequent treatment. Second, for patients in whom no surgical options are advisable, systematic treatment, such as chemotherapy and cellular immunotherapy, based on one tumor may not be effective for tumor on the contralateral side and its derivatives. Third, although bilateral carcinomas may harbor distinctive somatic mutations, the tumor evolution trajectories converge on a limited number of signaling pathways. Drugs targeting the shared pathways may be more effective for systematic treatment of these patients.

## Methods

### Samples

Three male patients and one female patient with bilateral ccRCC were involved in this study approved by the Ethics Committee of Beijing Friendship Hospital (register number BJFH-EC/2013-079). All procedures were carried out in accordance with the approved guidelines. Written informed consents were obtained from all four patients. All cases were sporadic with no known family history. No renal cysts or tumors in other organs, such as retinal and brain, were detected, ruling out the possibility of VHL syndrome. Partial nephrectomy was performed at two stages, three months apart, to remove the tumors (one at each stage). Subsequent histopathological analysis confirmed all eight tumors as ccRCC (Fig. 1 and Supplementary Figure 3). Fresh frozen or paraffin-embedded tissue samples (0.5–0.8 cm each) were obtained and processed at the time of nephrectomy. For patient BC_1, fresh frozen tumor sample BC_1L and peritumoral tissue BC_1P (normal peritumoral tissue) were taken from left kidney. The right tumor sample BC_1R was paraffin-embedded. For the patient BC_2, right tumor sample BC_2R and the peritumoral sample BC_2P were freshly frozen. The left tumor sample BC_2L was paraffin-embedded. For patients BC_3 and BC_4, all samples were freshly frozen. BC_3R1, BC_3R2, BC_3R3 and BC_3R4 were taken from four distinct regions in the right tumor of patient BC_3, representing the spatial extent of this tumor and its morphological heterogeneity. BC_4R1 and BC_4R2 were dissected from two distinct regions in the right tumor of patient BC_4. Normal peritumoral tissues were taken as reference for each tumor (BC_3RP, BC_3LP, BC_4RP and BC_4LP). For each tumor, a matched sample was taken for histopathological assessment. At the time of this report, all four patients remain in complete remission.

### Whole exome sequencing

Genomic DNA was extracted from tissue samples using the Qiagen DNeasy kit according to the manufacturer’s protocol. WES was performed for DNA samples from patient BC_1 and BC_2. The Genewiz, Inc. (Beijing) performed initial quality control assessments and subsequent exome capture using the Illumina TruSeq Exome Enrichment kit. All samples were paired-end multiplex sequenced (2 × 100) on the Illumina Hiseq 2500 platform to a median target depth of over 50×. WES data have been deposited in NCBI under BioProject ID PRJNA295344.

### Processing of sequencing data

Paired-end reads from the Genewiz, Inc. (Beijing) underwent quality control before alignment to the reference human genome (hg19) using Burrows-Wheeler alignment (BWA, version 0.6.2-r126) and SAMtools (version 0.1.18 (r982:295)). Duplicate reads were removed with tools in SAMtools. Realignment and recalibration were performed using the Genome Analysis Toolkit (GATK, version 2.3–9-ge5ebf34). Single nucleotide variants (SNVs) and indels were called using GATK with default setting. Variant calls were filtered (each allele with 5≤ depth ≤ 150, combined depth of reference allele and alternative allele ≥15) to remove variants in poorly aligned reads or with sequencing errors. Known high frequency SNVs and indels were filtered using the dbSNP and 1000 g. Somatic SNVs and indels in tumor samples were detected using the corresponding normal tissue as reference. PCR and Sanger sequencing was performed for a total of 11 variant alleles (each for all three samples in the corresponding patient) to validate WES and calling accuracy.

### SCNA profile analysis

Raw copy number estimates of tumor samples were generated from the WES data using VarScan2 with default settings^31^. Low mapability regions and the sex chromosomes were excluded. After adjusting log_2_ values for GC content, a circular binary segment (CBS) algorism in the DNAcopy package was applied to delineate segments by copy number (indicated as redline) and to identify significant change-points^31^,^32^. Adjacent segments of similar copy number were subsequently merged. Segments that encompass ≥25% of a chromosome arm were considered as large-scale; otherwise were classified as focal events^31^.

### Evaluation of mutations on protein function

Protein variation effect analyzer (PROVEAN) was used to estimate effects of SNVs and indels on protein function. Mutations with a score less than −4.1 (probability >90.3%, sensitivity 57.6%) were considered deleterious^33^.

### Bisulfite modification, PCR and pyrosequencing of DNA

For bisulfite conversion, 1.2 μg of genomic DNA was bisulfite treated using EpiTect Bisulfite kit (QIAGEN) according to the User’s Manual. Bisulfite conversion was performed in a thermal cycler using the specified cycling conditions. Modified DNA was bound to the EpiTect spin columns, washed twice, desulfonated and then eluted in 20 μl volume. The bisulfite converted DNA was either used immediately or stored at −20 °C.

PCR amplification of bisulfite converted DNA was performed using PyroMark PCR kit (QIAGEN). Primers for PCR amplification were designed using Methyl Primer Express v1.0 (Supplementary Table 8). All primers were located within CpG islands in the promoter region of *VHL*. The PCR reaction used the following conditions: 95 °C for 15 min, followed by 45 cycles of: (95 °C 30 s, 60 °C 30 s, 72 °C 30 s), and 72 °C for 10 min.

Pyrosequencing was performed to assess the methylation level of *VHL* promoter region using the PyroMark Q96 ID and PyroMark Gold Reagent Kit (QIAGEN) according to the User’s Manual. Primers for pyrosequencing were designed using Pyromark assay design (Supplementary Table 8). 50 μl of the PCR product was added to a total reaction volume of 50 μl containing binding buffer and sepharose beads. The reaction mix was agitated for 10 min at room temperature in order to facilitate the attachment of beads to the biotinylated single strand DNA. The biotinylated DNA was then released to the PyroMark plate well each containing sequencing primer with annealing buffer in a reaction volume of 45 μl. The mixture was denatured for 2 min at 80 °C and re-annealed at room temperature. The plate was put in the PyroMark Q96 ID and run with relevant run file. Subsets of cases were re-analyzed with repeated PCRs and subsequent Pyrosequencing to validate the initial results.

### Pathway analysis

Total of 47 genes are annotated in pathways. Information about gene annotation was obtained from the Kyoto Encyclopedia of Genes and Genomes (KEGG) and Reactome. The mutant genes were also tested in STRING to predicted protein interactions.

## Additional Information

**How to cite this article**: Ji, Z. *et al*. Independent Tumor Origin in Two Cases of Synchronous Bilateral Clear Cell Renal Cell Carcinoma. *Sci. Rep.* **6**, 29267; doi: 10.1038/srep29267 (2016).

## Supplementary Material

Supplementary Information
