# Supplementary material for: Independent Tumor Origin in Two Cases of Synchronous Bilateral Clear Cell Renal Cell Carcinoma
Source: Sci Rep. 2016 Jul 7;6:29267. doi: 10.1038/srep29267 (PMC4935960; doi:10.1038/srep29267)
Supplement: Supplementary Information [file srep29267-s1.pdf]

Independent Tumor Origin in Two Cases of Synchronous Bilateral Clear Cell Renal Cell Carcinoma

Zhengguo Ji[1,2,*], Jialu Zhao[1,*], Tian Zhao[1], Yuying Han[1], Yujun Zhang[4] & Haihong Ye[1,3]

[1]Department of Medical Genetics and Developmental Biology, School of Basic Medical Sciences. [2]Department of Urology, Beijing Friendship Hospital, [3]Beijing Institute for Brain Disorders, Center of Schizophrenia, Capital Medical University, Beijing 100069, China. [4]Institute of Chinese Materia Medica, Chinese Academy of Chinese Medical Science, Beijing 100700, China. *These authors contributed equally to this work. Correspondence and requests for materials should be addressed to H.Y (email: yehh@ccmu.edu.cn), Y.Z (email: yujun_zhang@vip.sina.com) or Y.H (cherry3291@163.com)

Supplementary Table 1. Clinical characteristics.

Case	Age at diagnosis (yr)	Gender	Side	Fuhrman grade	TNM stage	Tumor ID
BC_1	63	M	Left	G1	T1aN0M0	BC_1L
			Right	G1	T1aN0M0	BC_1R
BC_2	52	M	Left	G1	T1bN0M0	BC_2L
			Right	G1	T1bN0M0	BC_2R
BC_3	57	F	Left	G1	T1aN0M0	BC_3L
			Right	G2	T2bN0M0	BC_3R
BC_4	57	M	Left	G1	T1aN0M0	BC_4L
			Right	G3	T3aN0M0	BC_4R

Supplementary Table 2. Statistics of whole exome sequencing data.

Case	Raw variants		After database filter		Exome variants	
	SNVs	Indels	SNVs	Indels	SNVs	Indels
BC_1	100,607	13,754	1,638	1,878	360	53
BC_2	99,176	13,516	1,673	1,876	352	49

Supplementary Table 3. Sanger sequencing of 11 variants detected in tumors. For each variant allele Sanger sequencing was performed for both tumors and the normal tissue. WES data show that all three tissues in BC_2 harbor a G/A mutation in *NAV3*. However, this mutation in BC_2L was not validated in Sanger sequencing. All other variants were confirmed in Sanger sequencing. Mutation sites are marked by asterisks in images of a chromatogram section. R: the sequencing direction is reverse of the sequence from the database.

Illumina data			Sanger sequencing data		
Gene name	position	Ref/Tumor	BC_1P	BC_1R	BC_1L
VHL	Chr3 10188203	AG/A	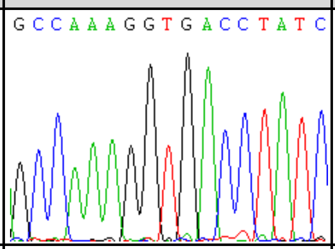	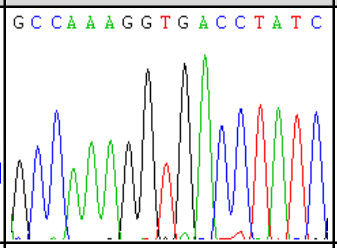	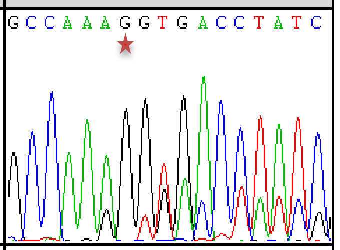
KDM5C	ChrX 53222393	T/C	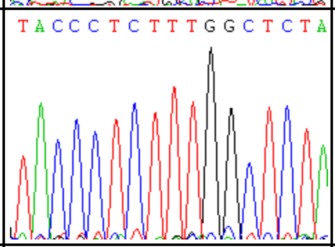	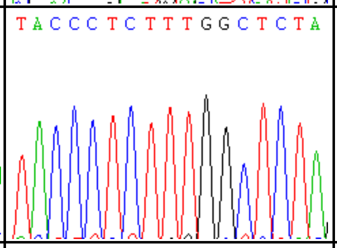	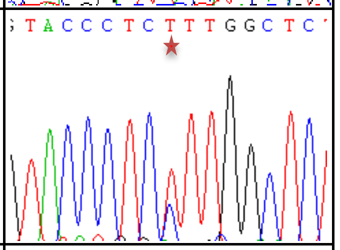
ZFHX4	Chr8 77618767	C/T	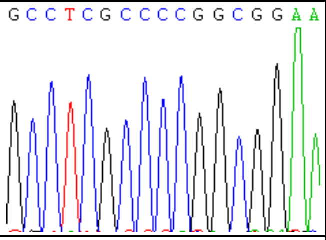	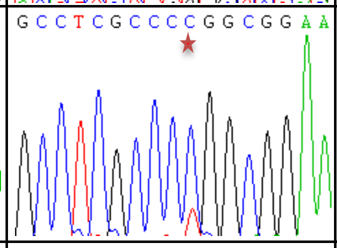	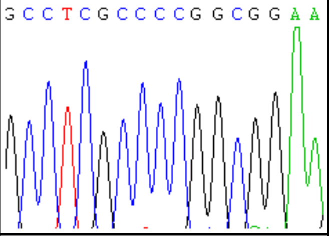
TGS1	Chr8 56699253	C/T	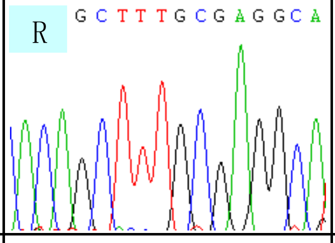	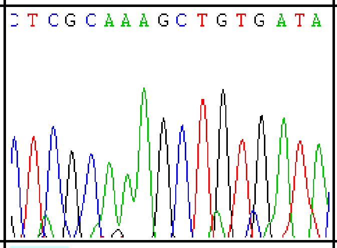	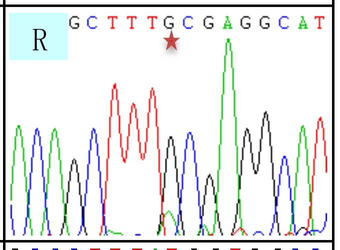
TCF21	Chr6 13421286 5	G/A	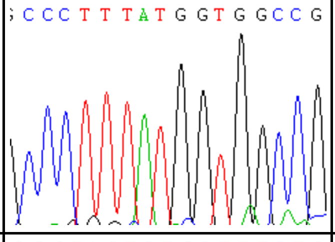	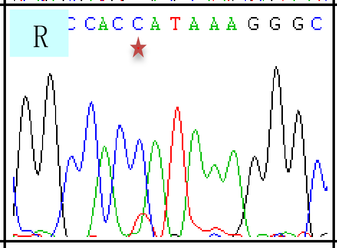	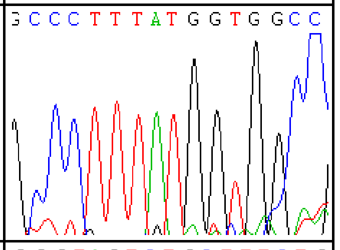
PTCH1	Chr9 98229527	C/A	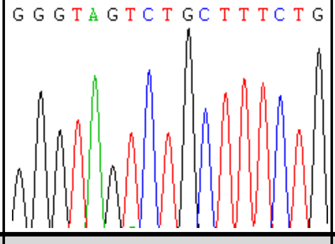	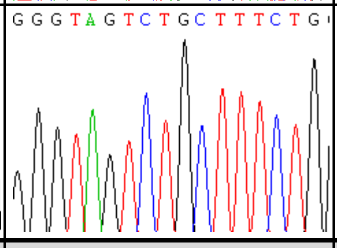	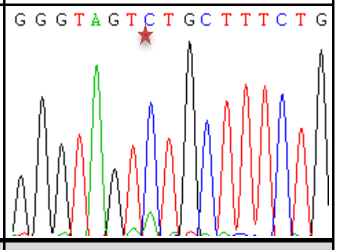
Gene name	position	Ref/Tumor	BC_2P	BC_2R	BC_2L
VHL	Chr3 10188223	CT/C	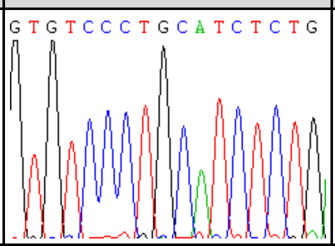	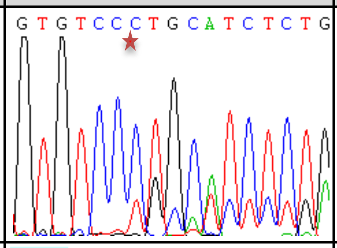	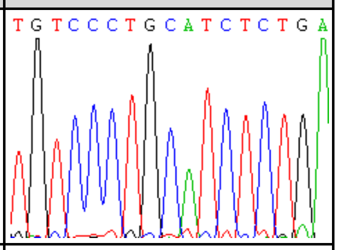
PTEN	Chr10 89720757	TG/T	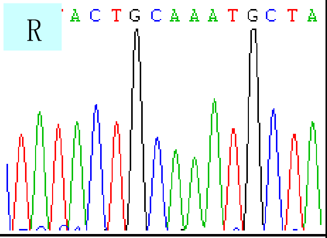	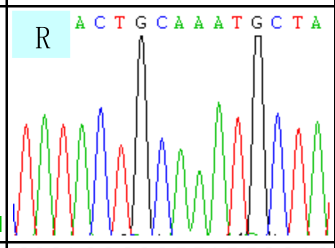	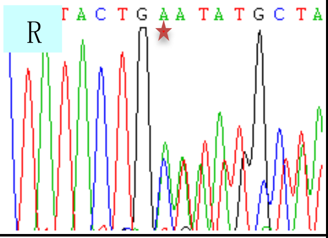
GRB7	Chr17 37902223	G/A	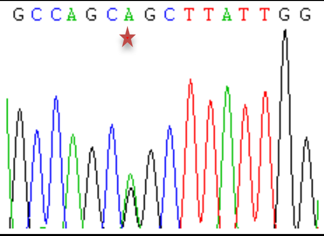	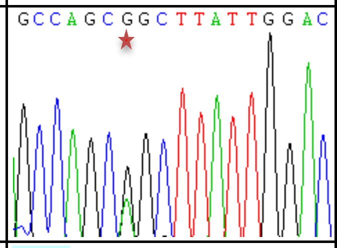	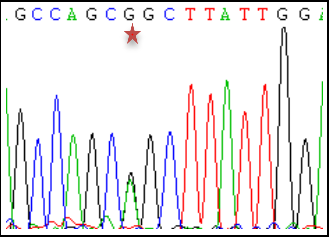
AKAP13	Chr15 86124462	G/A	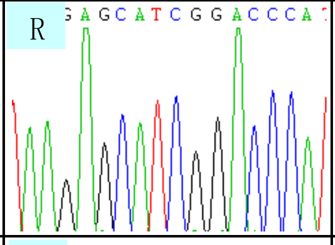	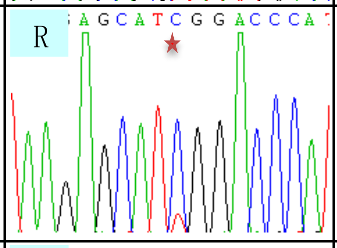	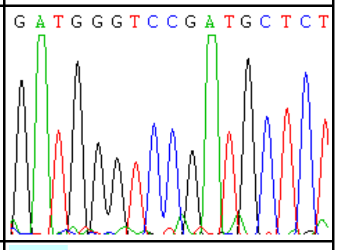
NAV3	Chr12 78400783	G/A	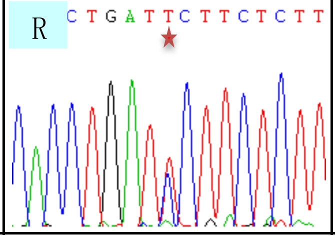	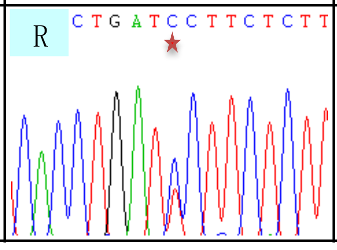	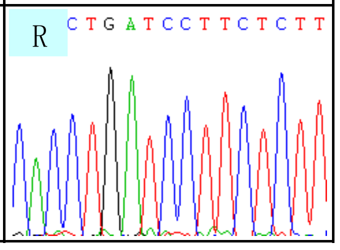

Supplementary Table 4. Non-synonymous and slicing site somatic mutations in four tumors.

Tumor ID	Type	Chromsome	Position	Alleles (Ref/Tumour)	Consequence type	Ensembl gene ID	Gene name	Comments
BC_1L	SNV	Chr1	1.67E+08	G/C	Non-synonymous SNV	ENSG00000198842	*DUSP27*	
BC_1L	SNV	Chr1	2.28E+08	G/T	Non-synonymous SNV	ENSG00000143774	*GUK1*	
BC_1L	SNV	Chr2	15769799	C/T	Non-synonymous SNV	ENSG00000079785	*DDX1*	
BC_1L	SNV	Chr2	37232894	T/C	Non-synonymous SNV	ENSG00000008869	*HEATR5B*	
BC_1L	Indel	Chr3	10188203	CT/C	Frameshift deletion	ENSG00000134086	*VHL*	
BC_1L	SNV	Chr5	44388494	C/A	Non-synonymous SNV	ENSG00000070193	*FGF10*	
BC_1L	SNV	Chr5	1.31E+08	A/C	Non-synonymous SNV	ENSG00000158987	*RAPGEF6*	
BC_1L	SNV	Chr8	28573665	C/T	Non-synonymous SNV	ENSG00000012232	*EXTL3*	
BC_1L	SNV	Chr8	56699253	C/T	Gain of stop codon	ENSG00000137574	*TGS1*	
BC_1L	SNV	Chr8	1.46E+08	G/C	Non-synonymous SNV	ENSG00000167701	*GPT*	
BC_1L	SNV	Chr9	98229527	C/A	Non-synonymous SNV	ENSG00000185920	*PTCH1*	
BC_1L	SNV	Chr12	65116826	A/C	Non-synonymous SNV	ENSG00000135677	*GNS*	
BC_1L	Indel	Chr12	88588899	A/AT	Frameshift insertion	ENSG00000139324	*TMTC3*	
BC_1L	SNV	Chr13	46936727	C/G	Non-synonymous SNV	ENSG00000102445	*KIAA0226L*	
BC_1L	SNV	Chr15	66793749	C/A	Non-synonymous SNV	ENSG00000174444	*RPL4*	
BC_1L	SNV	Chr17	30510253	C/A	Non-synonymous SNV	ENSG00000126858	*RHOT1*	

BC_1L	SNV	Chr18	51820307	T/A	Non-synonymous SNV	ENSG00000101751	*POLI*
BC_1L	SNV	Chr20	10394580	C/G	Splicing site SNV	ENSG00000125863	*MKKS*
BC_1L	SNV	Chr20	34242438	G/T	Non-synonymous SNV	ENSG00000244462	*RBM12*
BC_1L	SNV	Chr22	18900980	C/T	Non-synonymous SNV	ENSG00000100033	*PRODH*
BC_1L	SNV	ChrX	53222393	T/G	Non-synonymous SNV	ENSG00000126012	*KDM5C*
BC_1R	SNV	Chr3	46718442	C/T	Non-synonymous SNV	ENSG00000178038	*ALS2CL*
BC_1R	SNV	Chr4	265901	A/G	Non-synonymous SNV	ENSG00000186777	*ZNF732*
BC_1R	SNV	Chr6	1.34E+08	G/A	Non-synonymous SNV	ENSG00000118526	*TCF21*
BC_1R	SNV	Chr7	63726834	C/A	Non-synonymous SNV	ENSG00000197123	*ZNF679*
BC_1R	SNV	Chr8	77618767	C/T	Non-synonymous SNV	ENSG00000091656	*ZFHX4*
BC_1R	SNV	Chr9	33674640	G/T	ncRNA SNV	ENSG00000237984	*PTENP1*
BC_1R	SNV	Chr10	15089331	C/A	Non-synonymous SNV	ENSG00000152463	*OLAH*
BC_1R	SNV	Chr15	76165799	A/G	Non-synonymous SNV	ENSG00000140367	*UBE2Q2*
BC_1R	SNV	Chr16	84215998	G/A	Non-synonymous SNV	ENSG00000103168	*TAF1C*
BC_1R	SNV	Chr17	42476660	A/T	Non-synonymous SNV	ENSG00000186566	*GPATCH8*
BC_1R	SNV	Chr19	50391551	C/A	Non-synonymous SNV	ENSG00000104946	*TBC1D17*
BC_1R	SNV	Chr20	60293915	G/C	Non-synonymous SNV	ENSG00000179253	*RP11-429E11.3*
BC_2L	SNV	Chr1	9305578	G/T	Non-synonymous SNV	ENSG00000049239	*H6PD*
BC_2L	SNV	Chr1	1.59E+08	G/A	Non-synonymous SNV	ENSG00000186440	*OR6P1*

BC_2L	SNV	Chr2	61390082	C/G	Non-synonymous SNV	ENSG00000237651	*C2orf74*
BC_2L	SNV	Chr2	1.7E+08	C/A	Non-synonymous SNV	ENSG00000073734	*ABCB11*
BC_2L	SNV	Chr2	1.98E+08	T/C	Non-synonymous SNV	ENSG00000115540	*MOB4*
BC_2L	SNV	Chr2	2.42E+08	A/G	Non-synonymous SNV	ENSG00000115685	*PPP1R7*
BC_2L	SNV	Chr3	9515202	G/T	Gain of stop codon	ENSG00000168137	*SETD5*
BC_2L	SNV	Chr4	56847460	G/A	Non-synonymous SNV	ENSG00000174799	*CEP135*
BC_2L	SNV	Chr4	1.04E+08	C/G	Non-synonymous SNV	ENSG00000138778	*CENPE*
BC_2L	SNV	Chr4	1.46E+08	A/T	Non-synonymous SNV	ENSG00000170365	*SMAD1*
BC_2L	SNV	Chr5	1.37E+08	T/C	Non-synonymous SNV	ENSG00000177733	*HNRNPA0*
BC_2L	SNV	Chr5	1.41E+08	A/T	Non-synonymous SNV	ENSG00000113209	*PCDHB5*
BC_2L	SNV	Chr6	86303334	G/C	Non-synonymous SNV	ENSG00000135317	*SNX14*
BC_2L	SNV	Chr6	1.63E+08	C/A	Non-synonymous SNV	ENSG00000185345	*PARK2*
BC_2L	SNV	Chr7	31682945	C/G	Non-synonymous SNV	ENSG00000180347	*CCDC129*
BC_2L	SNV	Chr9	33116007	T/A	Non-synonymous SNV	ENSG00000086062	*B4GALT1*
BC_2L	SNV	Chr10	13222576	T/G	Non-synonymous SNV	ENSG00000065328	*MCM10*
BC_2L	Indel	Chr10	89720759	TG/T	Frameshift deletion	ENSG00000171862	*PTEN*
BC_2L	SNV	Chr10	89720757	T/A	Non-synonymous SNV	ENSG00000171862	*PTEN*
BC_2L	SNV	Chr11	47505991	C/A	Non-synonymous SNV	ENSG00000149187	*CELF1*
BC_2L	SNV	Chr11	73073780	G/C	Splicing site SNV	ENSG00000110237	*ARHGEF17*

BC_2L	SNV	Chr11	1.19E+08	C/A	Non-synonymous SNV	ENSG00000036672	*USP2*	
BC_2L	SNV	Chr12	69653839	C/A	Non-synonymous SNV	ENSG00000111605	*CPSF6*	
BC_2L	SNV	Chr14	59113294	C/A	Non-synonymous SNV	ENSG00000165617	*DACT1*	
BC_2L	SNV	Chr14	1.07E+08	C/T	Non-synonymous SNV	ENSG00000211953	*IGHV3-30*	Shared with BC_2R
BC_2L	SNV	Chr16	1496338	C/A	Non-synonymous SNV	ENSG00000103249	*CLCN7*	
BC_2L	SNV	Chr17	8273500	T/G	Non-synonymous SNV	ENSG00000184619	*KRBA2*	
BC_2L	SNV	Chr17	37902223	G/A	Non-synonymous SNV	ENSG00000141738	*GRB7*	Shared with BC_2R and BC_2P
BC_2L	Indel	Chr17	80445848	CTGCTGCGGC AGA/C	Non-frameshift deletion	ENSG00000141562	*NARF*	
BC_2L	SNV	Chr18	74963016	C/A	Non-synonymous SNV	ENSG00000166573	*GALR1*	
BC_2L	SNV	Chr19	2414017	G/A	Splicing site SNV	ENSG00000178297	*TMPRSS9*	
BC_2L	SNV	Chr19	23927334	A/G	Non-synonymous SNV	ENSG00000196172	*ZNF681*	
BC_2L	SNV	Chr21	38516859	A/T	Gain of stop codon	ENSG00000182670	*TTC3*	
BC_2L	SNV	Chr22	50167466	C/T	Splicing site SNV	ENSG00000100425	*BRD1*	
BC_2R	SNV	Chr1	39913497	G/A	Non-synonymous SNV	ENSG00000127603	*MACF1*	
BC_2R	SNV	Chr2	71043840	C/T	Non-synonymous SNV	ENSG00000152672	*CLEC4F*	
BC_2R	Indel	Chr3	10188223	AG/A	Frameshift deletion	ENSG00000134086	*VHL*	
BC_2R	SNV	Chr4	56283277	T/A	Non-synonymous SNV	ENSG00000134851	*TMEM165*	
BC_2R	SNV	Chr4	1.48E+08	C/T	Non-synonymous SNV	ENSG00000151617	*EDNRA*	
BC_2R	SNV	Chr5	5465339	G/C	Non-synonymous SNV	ENSG00000164151	*KIAA0947*	

BC_2R	SNV	Chr5	1.38E+08	C/G	Non-synonymous SNV	ENSG00000146006	*LRRTM2*	
BC_2R	SNV	Chr6	35888056	G/T	Non-synonymous SNV	ENSG00000096063	*SRPK1*	
BC_2R	SNV	Chr6	1.08E+08	C/T	Non-synonymous SNV	ENSG00000146285	*SCML4*	
BC_2R	SNV	Chr7	1.01E+08	G/C	Non-synonymous SNV	ENSG00000128564	*VGF*	
BC_2R	SNV	Chr8	27964240	T/A	Non-synonymous SNV	ENSG00000134014	*ELP3*	
BC_2R	SNV	Chr8	87443664	T/C	Non-synonymous SNV	ENSG00000123124	*WWP1*	
BC_2R	SNV	Chr9	35108229	C/T	Non-synonymous SNV	ENSG00000005238	*FAM214B*	
BC_2R	SNV	Chr9	75435790	G/A	Non-synonymous SNV	ENSG00000165091	*TMC1*	
BC_2R	SNV	Chr11	62591713	G/T	Gain of stop codon	ENSG00000162236	*STX5*	
BC_2R	SNV	Chr12	72289834	G/C	Non-synonymous SNV	ENSG00000121749	*TBC1D15*	
BC_2R	SNV	Chr12	78400783	G/A	Non-synonymous SNV	ENSG00000067798	*NAV3*	Shared with BC_2P
BC_2R	SNV	Chr14	1.07E+08	C/T	Non-synonymous SNV	ENSG00000211953	*IGHV3-30*	Shared with BC_2L
BC_2R	SNV	Chr14	37050561	A/C	Non-synonymous SNV	ENSG00000136327	*NKX2-8*	Shared with BC_2P
BC_2R	SNV	Chr15	86124462	G/A	Non-synonymous SNV	ENSG00000170776	*AKAP13*	
BC_2R	SNV	Chr16	11859487	C/T	Non-synonymous SNV	ENSG00000122299	*ZC3H7A*	
BC_2R	SNV	Chr17	12920229	T/C	Non-synonymous SNV	ENSG00000006744	*ELAC2*	
BC_2R	SNV	Chr17	31261308	G/A	Splicing site SNV	ENSG00000006042	*TMEM98*	
BC_2R	SNV	Chr17	37902223	G/A	Non-synonymous SNV	ENSG00000141738	*GRB7*	Shared with BC_2L and BC_2P
BC_2R	SNV	Chr17	80895792	G/C	Non-synonymous SNV	ENSG00000141556	*TBCD*	

BC_2R	SNV	Chr19	55994851	T/A	Non-synonymous SNV	ENSG00000197483	*ZNF628*
BC_2R	SNV	Chr22	51010505	C/G	Non-synonymous SNV	ENSG00000205560	*CPT1B*
BC_2R	SNV	ChrX	1.14E+08	T/A	Non-synonymous SNV	ENSG00000130224	*LRCH2*

Supplementary Table 5. Mutations in ccRCC- and other cancer-related genes detected in tumor samples. * indicates deleterious mutations.

Case	Gene name	Tumor ID	Position	Mutation type	DNA change	AA change	PROVEAN score
BC_1	*VHL*	BC_1L	Chr3 10188203	Frameshift deletion	CT/C	W117 fs	-252.20 *
	KDM5C	BC_1L	ChrX 53222393	SNV	A>G	K1480R	-1.77
	FGF10	BC_1L	Chr5 44388494	SNV	G>T	K97N	-1.15
	TGS1	BC_1L	Chr8 56699253	SNV	C>T	Q266X	-2.20
	PTCH1	BC_1L	Chr9 98229527	SNV	G>T	D811Y	-3.11
	TCF21	BC_1R	Chr6 13421286	SNV	G>A	M155I	-0.34
	ZFHX4	BC_1R	Chr8 77618767	SNV	C>T	P815L	-7.55 *
	PTENP1	BC_1R	Chr9 33674640	SNV/ncRNA	G>T	-	-
BC_2	*PTEN*	BC_2L	Chr10 89720759	Frameshift deletion	TG/T	C304 fs	-163.22 *
			Chr10 89720757	SNV	T>A	I303N	-1.06
	VHL	BC_2R	Chr3 10188223	Frameshift deletion	AG/A	T124 fs	-225.47 *
	AKAP13	BC_2R	Chr15 86124462	SNV	G>A	D1955N	-0.38
	GRB7	BC_2P+L+R	Chr17 37902223	SNV	G>A	R443Q	-2.78
	NAV3	BC_2P+R	Chr12 78400783	SNV	G>A	D489N	-1.20

Supplementary Table 6. Sanger sequencing of two patients with metachronous bilateral ccRCCs. Mutation sites are marked by asterisks in images of a chromatogram section. All the sequencing direction is reverse of the sequence from the database.

VHL			Sanger sequencing data						
	Position	Varients	BC_3RP	BC_3R1	BC_3R2	BC_3R3	BC_3R4	BC_3LP	BC_3L
Case 3	10188309	T→G transversion	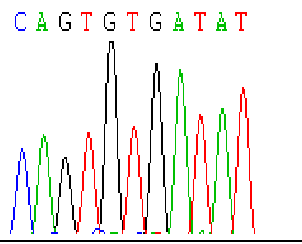	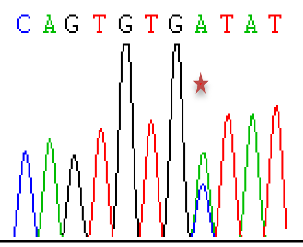	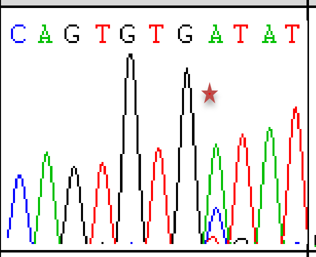	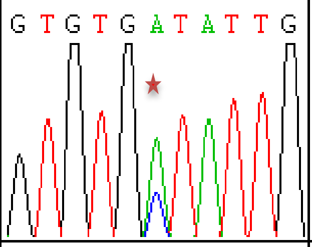	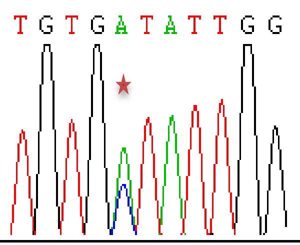	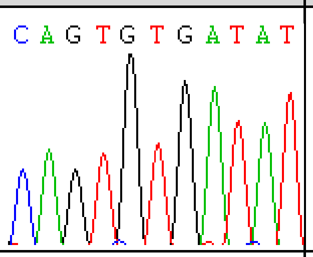	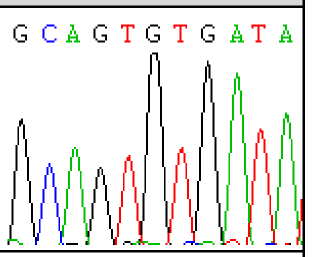
	10191645 - 10191655	ATTGAAGATTT deletion	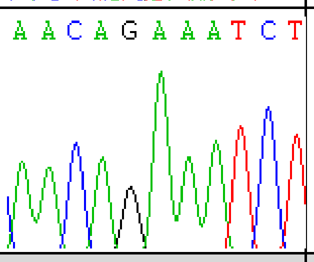	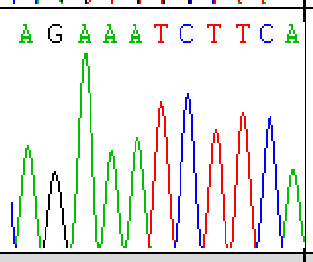	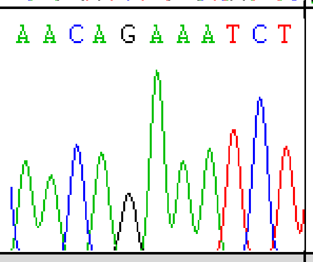	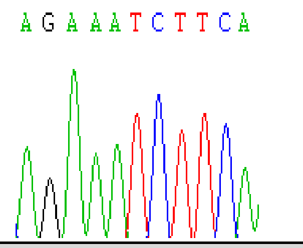	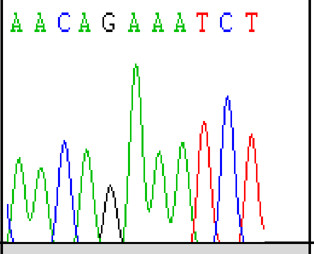	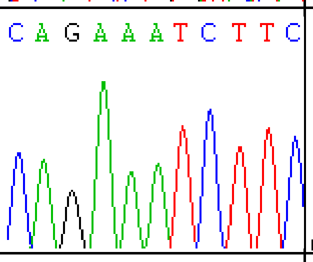	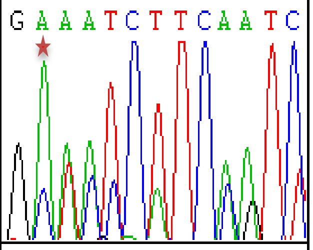
	Position	Varients	BC_4RP	BC_4R1	BC_4R2	BC_4LP	BC_4L		
Case 4	10183800 - 10183801	TA insertion	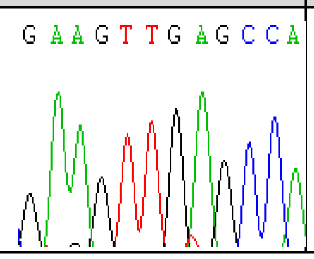	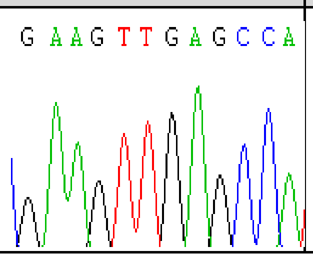	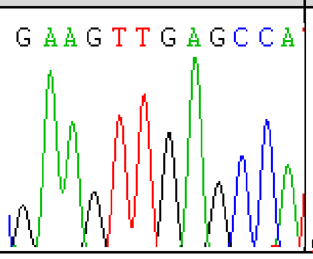	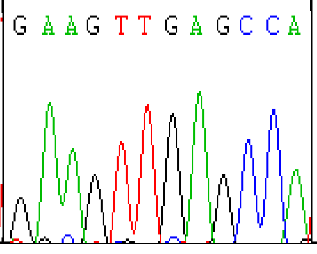	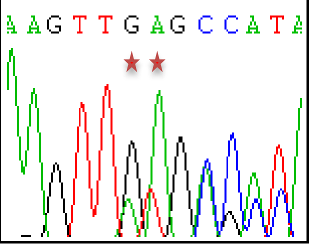		

Supplementary Table 7. KEGG pathway analysis

Tumor ID	Pathway	Gene
BC_1R	04120 Ubiquitin mediated proteolysis (1)	*UBE2Q2*
	00061 Fatty acid biosynthesis (1)	*OLAH*
BC _1L	01200 Carbon metabolism (1)<br>01210 2-Oxocarboxylic acid metabolism (1)<br>01230 Biosynthesis of amino acids (1)<br>00710 Carbon fixation in photosynthetic organisms (1)<br>00250 Alanine, aspartate and glutamate metabolism (1)	*GPT*
	00230 Purine metabolism (1)	*GUK1*
	00330 Arginine and proline metabolism (1)	*PRODH*
	00534 Glycosaminoglycan biosynthesis - heparan sulfate / heparin (1)	*EXTL3*
	04142 Lysosome (1)<br>00531 Glycosaminoglycan degradation (1)	*GNS*
	03010 Ribosome (1)	*RPL4*
	03013 RNA transport (1)	*TGS1*
	03460 Fanconi anemia pathway (1)	*POLI*
	04014 Ras signaling pathway (1)<br>04015 Rap1 signaling pathway (2)<br>04010 MAPK signaling pathway (1)<br>04151 PI3K-Akt signaling pathway (1)<br>04810 Regulation of actin cytoskeleton (1)<br>05200 Pathways in cancer (2)<br>05218 Melanoma (1)	*FGF10*
	04015 Rap1 signaling pathway (2)	*RAPGEF6*
	04340 Hedgehog signaling pathway (1)<br>04024 cAMP signaling pathway (1)<br>05200 Pathways in cancer (3)<br>05205 Proteoglycans in cancer (1)<br>05217 Basal cell carcinoma (1)	*PTCH1*
	04120 Ubiquitin mediated proteolysis (1)<br>05211 Renal cell carcinoma (1)	*VHL*

	05200 Pathways in cancer (3)	
	03050 Proteasome (1) 05169 Epstein-Barr virus infection (1)	*PSMD8*
BC_2R	01212 Fatty acid metabolism (1)<br>00071 Fatty acid degradation (1)<br>04152 AMPK signaling pathway (1)<br>04920 Adipocytokine signaling pathway (1)<br>03320 PPAR signaling pathway (1)	*CPT1B*
	03013 RNA transport (1)	*ELAC2*
	04130 SNARE interactions in vesicular transport (1)	*STX5*
	04120 Ubiquitin mediated proteolysis (2)	*VHL WWP1*
	04066 HIF-1 signaling pathway (1)	*VHL*
	05200 Pathways in cancer (3)	
	05211 Renal cell carcinoma (1)	
	04020 Calcium signaling pathway (1)<br>04024 cAMP signaling pathway (1)<br>04022 cGMP - PKG signaling pathway (1)<br>04080 Neuroactive ligand-receptor interaction (1)<br>04270 Vascular smooth muscle contraction (1)	*EDNRA*
	05168 Herpes simplex infection (1)	*SRPK1*
	00562 Inositol phosphate metabolism (1)<br>04068 FoxO signaling pathway (1)<br>04070 Phosphatidylinositol signaling system (1)<br>04151 PI3K-Akt signaling pathway (1)<br>04150 mTOR signaling pathway (1)<br>04115 p53 signaling pathway (1)<br>04510 Focal adhesion (1)<br>04530 Tight junction (1)<br>05230 Central carbon metabolism in cancer (1)<br>05206 MicroRNAs in cancer (1)<br>05214 Glioma (1)<br>05218 Melanoma (1)<br>05215 Prostate cancer (2)	*PTEN*

BC_2L	05213 Endometrial cancer (1)<br>05222 Small cell lung cancer (1)<br>05161 Hepatitis B (1)	
	00030 Pentose phosphate pathway (1)<br>01200 Carbon metabolism (1)	*H6PD*
	00052 Galactose metabolism (1)<br>00510 N-Glycan biosynthesis (1)<br>00513 Various types of N-glycan biosynthesis (1)<br>00514 Other types of O-glycan biosynthesis (1)<br>00533 Glycosaminoglycan biosynthesis - keratan sulfate (1)<br>00601 Glycosphingolipid biosynthesis - lacto and neolacto series (1)	*B4GALT1*
	03015 mRNA surveillance pathway (1)	*CPSF6*
	04141 Protein processing in endoplasmic reticulum (1)<br>04120 Ubiquitin mediated proteolysis (1)<br>05012 Parkinson's disease (1)	*PARK2*
	02010 ABC transporters (1)<br>04976 Bile secretion (1)	*ABCB11*
	04350 TGF-beta signaling pathway (1)<br>04390 Hippo signaling pathway (1)<br>04391 Hippo signaling pathway -fly (1)<br>04550 Signaling pathways regulating pluripotency of stem cells (1)<br>05202 Transcriptional misregulation in cancers (1)	*SMAD1*
	04080 Neuroactive ligand-receptor interaction (1)	*GALR1*
	04740 Olfactory transduction (1)	*OR6P1*

Supplementary Table 8. Primers used for PCR and Pyrosequencing of CpG islands in the VHL promoter region.

	Primers / Probes	Amplicon size (bp）
**Primers for PCR**	F-AATTGAGGTTTTTTGAGGTAGG<br>R-CCTAACCAACATAATAAAACCCC	279
**Probes for pyrosequencing**	PSQ1-AGGTTGGAGTGTAGTGG<br>SQ2-TTGAGTAGTTGGGATTATAG	
**Primers for PCR**	F-TTTTTTGAGTAGTTGGGATTATAGG<br>R-CATACCCATAAACCCAACACT	162
**Probes for pyrosequencing**	PSQ1-GGTTTYGAATTGTTGAT	

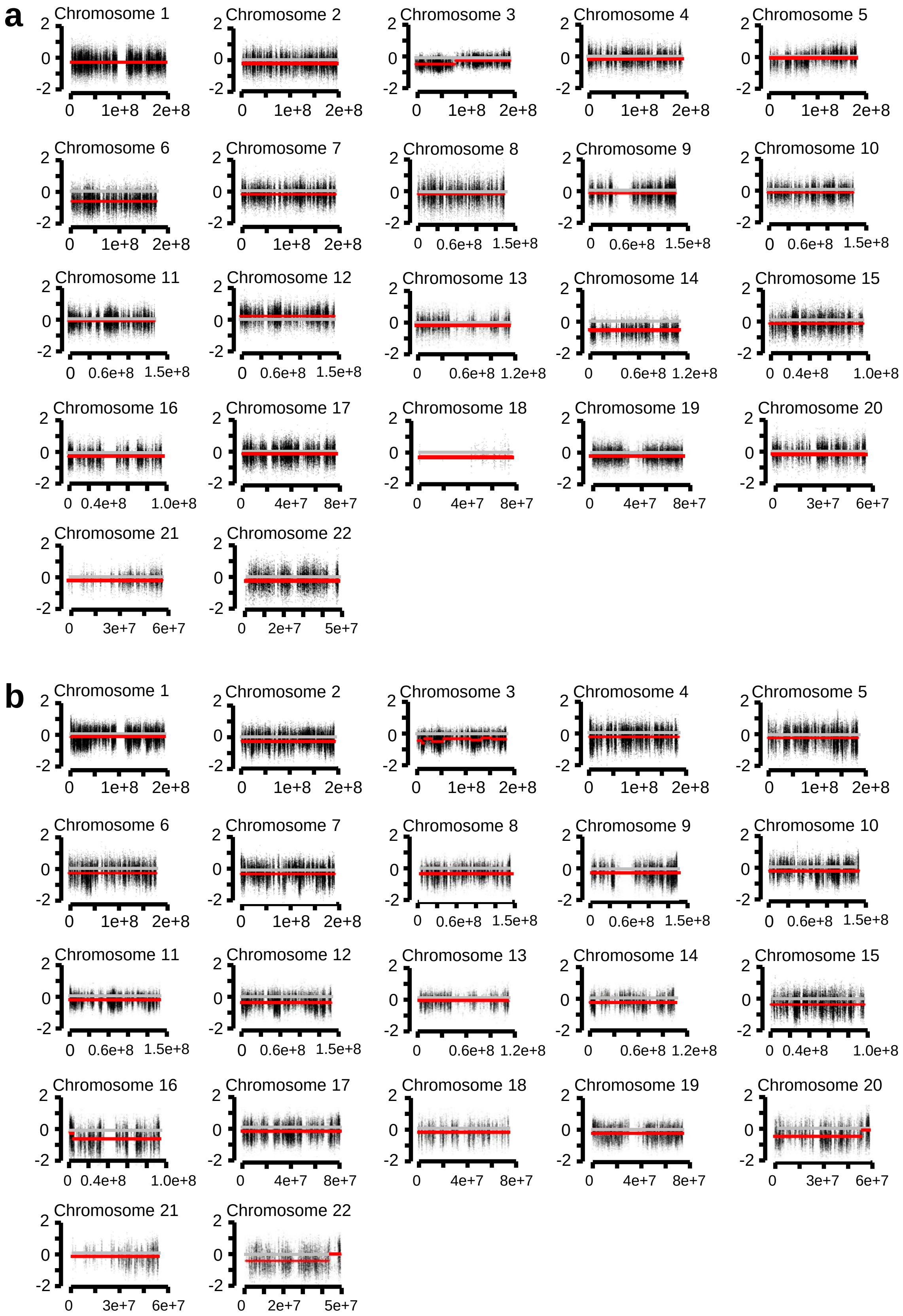

Supplementary figure 1. Somatic copy number analysis of tumor BC_1L (a) and BC_1R (b). Y-axis is $\log_2 R$, and the X-axis represents position along chromosomes.

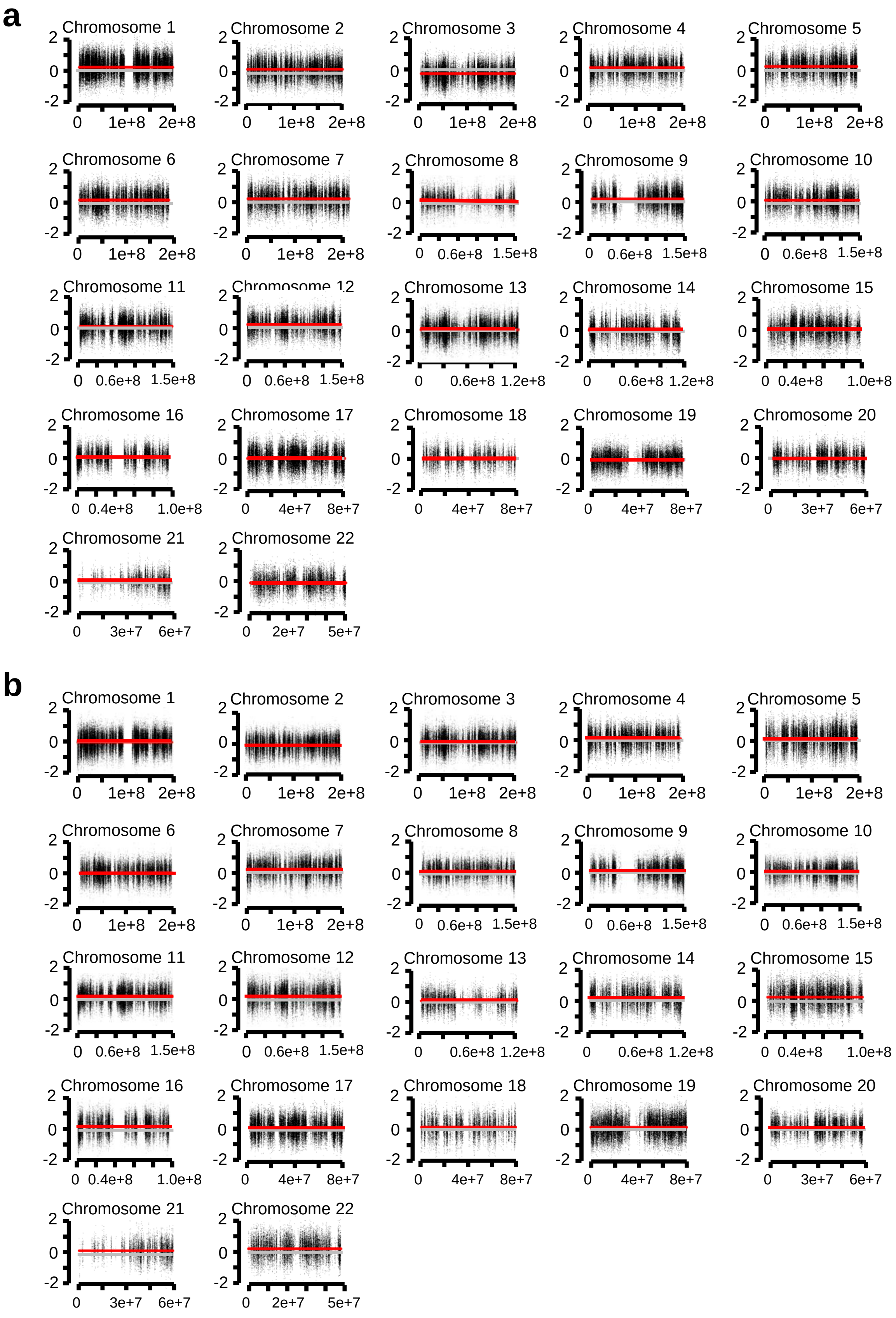

Supplementary figure 2. Somatic copy number analysis of tumor BC_2L (a) and BC_2R (b). Y-axis is $\log_2 R$, and the X-axis represents position along chromosomes.

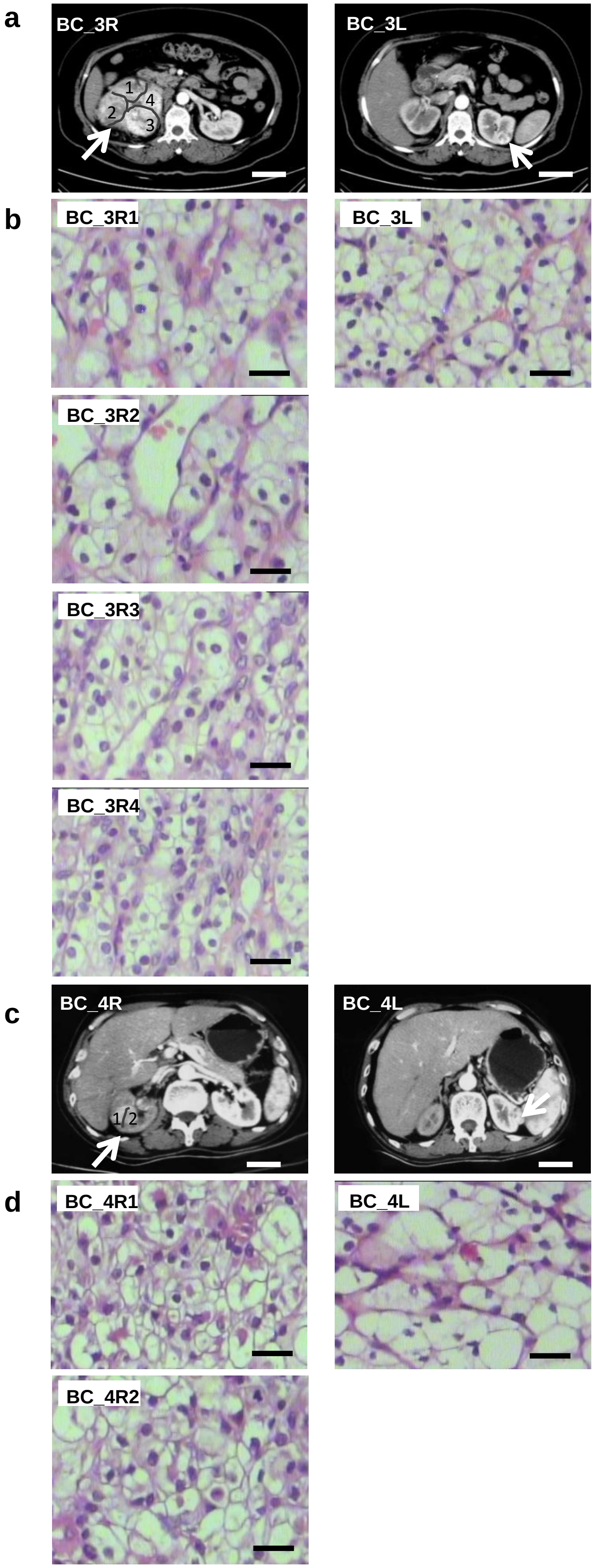

Supplementary figure 3. Imageology and histopathology information of the two metachronous bilateral ccRCC patients. (a) CT scan shows the tumor size of two kidneys in case BC_3. (b) Hematoxylin and eosin (HE) staining of four distinct regions in BC_3R (indicated as numbers in a) and BC_3L. (c) CT scan images of bilateral ccRCC in case BC_4. (d) HE staining of two distinct regions in case BC_4R (indicated as numbers in c) and BC_4L. Arrow, tumors. Scale bars in CT scan, 5 cm; scale bars in HE staining, 10 μm.
